# Cost effective assay choice for rare disease study designs

**DOI:** 10.1186/s13023-015-0226-9

**Published:** 2015-02-04

**Authors:** Desmond D Campbell, Robert M Porsch, Stacey S Cherny, Valeria Capra, Elisa Merello, Patrizia De Marco, Pak C Sham, Maria-Mercè Garcia-Barceló

**Affiliations:** Department of Psychiatry, University of Hong Kong, Hong Kong, SAR China; Centre for Genomic Sciences, University of Hong Kong, Hong Kong, SAR China; Department of Surgery, University of Hong Kong, Hong Kong, SAR China; State Key Laboratory of Brain and Cognitive Sciences, University of Hong Kong, Hong Kong, SAR China; Istituto Giannina Gaslini, Genoa, Italy

**Keywords:** WES, WGS, High-throughput assay, Rare disease, Study design

## Abstract

High throughput assays tend to be expensive per subject. Often studies are limited not so much by the number of subjects available as by assay costs, making assay choice a critical issue. We have developed a framework for assay choice that maximises the number of true disease causing mechanisms ‘seen’, given limited resources. Although straightforward, some of the ramifications of our methodology run counter to received wisdom on study design. We illustrate our methodology with examples, and have built a website allowing calculation of quantities of interest to those designing rare disease studies.

## Introduction

New technologies such as Next Generation Sequencing (NGS) have opened up new approaches for investigating the genetic aetiology of heritable diseases. For instance, a popular study design is whole genome or whole exome sequencing (WGS/WES) of affected offspring and their unaffected parents in order to identify *de novo* mutations and rare variants that could be potential risk factors for the disease. This has allowed investigation of diseases not previously amenable to genetic analyses due to their rarity, small family size and/or locus heterogeneity. For diseases following one of the classic Mendelian inheritance patterns, relatively small numbers of pedigrees are usually sufficient to reduce the number of candidate mutations to a manageable number [1]. Some combinations of mutations are sufficiently rare in unaffected individuals that their occurrence even in a single affected individual can provide evidence linking a gene to a disease. For instance, in whole genome sequencing of a Dutch population sample of 250 trios, only three rare loss of function compound heterozygous events were observed [2]. Such strategies have been used for both rare diseases and common multifactorial diseases, often with great success. Approximately 182 novel disease causing genes were found in the three years from 2009–12, predominantly using NGS in various small pedigree study designs [1]. Of the estimated 7,000 rare severe disorders, approximately half have now had risk genes identified for them. A caveat to this is that there is evidence that many reported pathogenic variants are in fact false positives [2,3].

Less commonly reported are the failures. Although some of these failures may be due to bad luck (e.g. uneven capture or coverage) or poor practice, many probably result from the aetiology of the disease under study being resistant to dissection by a particular study design. Matching of study design to disease aetiology is critical for a project’s success, but very difficult given that the aetiology is largely unknown. Other features that make study design difficult are the number and variety of assays available, and the typically high per subject cost of these assays. Researchers of rare diseases on constrained budgets may be limited not so much by the availability of disease cases as by assay costs. A choice has to be made on the most effective strategy; whether to spend the money on assaying a few individuals deeply or to spread the resources out shallow assaying more subjects. In this paper we lay down a framework for determining that strategy. Although straightforward, the results may run counter to received wisdom on study design.

Let us say we want to investigate the aetiology of a rare oligogenic disease. We may presume the heritability of the disease has already been justified e.g. in the application for funding to conduct the study. We can assume the disease is caused by a small number of causal genetic mutations/polymorphisms of large effect size in each case. However it is likely that there is little overlap in those risk mutations across cases, especially if the disease is phenotypically heterogeneous. Take Waardenburg syndrome (WS) as an example. This neurocristopathy (Neural Crest Cell disorder [4]) is characterised by the association of hearing loss with pigmentation abnormalities. Other abnormalities occurring in subsets of WS cases have given rise to its classification into sub-types. Six WS risk genes interact with each other in a melanocyte development related network. Although the risk genes associate with different subtypes of WS, there is much overlap regarding which genes cause which sub-types. There is also a lot of phenotypic heterogeneity within subtypes and incomplete penetrance within affected families. A few hundred point mutations have been identified in these WS associated genes based of their being predicted gene disruptive, most of these are private [5].

Using NGS or similar technologies to assay individual genomes it is typically possible to construct for any given case several plausible disease causing mechanisms, consistent with family history, conservation, predictions of mutation disruptiveness, etc. By disease causing mechanism we mean something very analogous to Rothman’s ‘sufficient cause’, i.e. a set of risk factors, none of which are sufficient in themselves to cause the disease, but which jointly guarantee that it will arise [6]. In our case the component causes are a set of mutations. The true causal mechanism for an individual will only be likely to be amongst the set of plausible candidate mechanisms if all the mutations that contribute to that mechanism have been observed via the assay(s) performed on the individual. Following construction of a set of candidate mechanisms for each of our cases, we can *then* look for commonality in these candidates across cases. It may be that a particular gene features in at least one of the candidate mechanisms of each case. That would make the gene a prime candidate for further investigation. Similarly there may be enrichment for genes involved in a particular function, a function that may be already implicated with the disease. For instance, there may be enrichment for insulin related genes, and having a diabetic mother may be a known disease risk factor. After identifying candidate disease mechanisms via a high throughput assay one would typically want to validate them via more precise assays, e.g. Sanger sequencing for genotype validation. Follow-up functional studies would be needed to validate any putative disease mechanisms identified.

High throughput assays may also be used in a clinical setting for the diagnosis of rare disease cases and for genetic counselling purposes. The costs/benefits of genetic testing for clinical purposes depend on many factors. One framework for evaluating their usefulness is the analytic validity, clinical validity, clinical utility and ethical, legal and social issues (ACCE) framework [7]. However here we are concerned with the efficient use of assays for research purposes in which case only the analytical validity of the assays is relevant.

## Methodology

Here we present methodology relevant to assay choice for two rare disease study scenarios. We have built a website implementing most of this methodology [8].

### Scenario 1: Assay choice for a new study

Typically the cost of high throughput assays is high and the funding available limited. Researchers may have many more cases available than they can afford to assay. The question addressed here is what is the most informative way to assay disease cases given a fixed budget, and an effectively unlimited number of cases?

We assume *no* overlap in the causal mutations across cases. Given this, the probability of the disease mechanism for a case being discoverable from a particular genetic assay on the individual is

1 $$ d={v}^c $$

where

- *d* = probability that all the relevant risk mutations for a particular case have been observed in the assays conducted on them
- *v* = hit rate = probability that a risk mutation for the disease will lie within the assayed loci
- *c* = complexity = the number of risk mutations needed to cause disease

Equation (1) is actually an approximation. It would be exact if all cases had exactly *c* causal mutations. For rare diseases the proportion of the cases that have more than *c* causal mutations is tiny and their effect on the value of *d* can be neglected.

The number of cases in which a complete disease mechanism has been observed will be binomially distributed

2 $$ m\sim Bin\left(d,n\right) $$

where

- *n* = number of cases assayed
- *m* = number of cases in which the complete disease mechanism has been observed, and hence is discoverable

The expected number of cases in which a complete disease mechanism has been observed is given by

$$ E\left[m\right]=nd $$

Substituting equation (1) into this gives

3 $$ E\left[m\right]=n{v}^c $$

In order to evaluate several alternative assays one needs to specify *n*, *c* and *v* for each assay and then calculate *E*[*m*] for each assay.

The cost effectiveness (utility) of a particular assay is given by

4 $$ U=E\left[m\right]/nP={v}^c/P $$

where

- *P* is the price per subject for the assay

We can see how assay utility relates to price and hit rate by setting the utility of two assays equal and rearranging thus

5 $$ \frac{P_2}{P_1}={\left(\frac{v_2}{v_1}\right)}^c $$

All of the above also applies to any set of assays considered as a single assay. For instance we could calculate the utility of a combination of WES and chip genotyping via equation (4).

In summary, the researcher is probably going to want to use the most cost effective assay on their cases (eqn. (4)), as this should maximise the number of completely observed disease causing mechanisms that can be seen for a fixed budget. Equation (3) gives the expectation of that number.

### Example 1

This is best illustrated by way of an example, and for that we will draw on our experience of a study of Caudal Regression Syndrome (CRS). CRS is a rare (1 in 10,000 births) congenital disorder characterized by abnormal development of the lower spine [9]. It is phenotypically heterogeneous; muscle hypotrophy resulting from lack of innervation may occur, as well as malformation of the pelvis and legs. Some CRS associated anomalies are seen in related clinical disorders, such as Currarino Syndrome [10] and VACTERL [11]. As with many congenital disorders, having a diabetic mother is a major risk factor for CRS. However the relative risk for CRS (=252) is the highest for all such disorders [12]. The underlying genetic basis of sporadic CRS is largely unknown [9,13,14].

We recently obtained funding to exome sequence four (*n* = 4) sporadic CRS cases and their unaffected parents. If we assumed

- Two risk mutations are needed to cause the disease, *c* = 2
- The hit rate provided by exome sequencing of a quarter, *v* = 0.27

then

$$ E\left[m\right]=n{v}^c=4{(0.27)}^2=0.29 $$

Alternatively we could have spent the money on genotyping the trios via a whole-genome genotyping microarray in order to look for rare and *de novo* Copy Number Variants (CNVs) (due to their popularity in Genomewide Association Studies such microarrays are often referred to as a GWAS chips). Let us assume

- the hit rate provided by CNVs detected though GWAS chip genotyping is *v* = 0.12
- GWAS chip genotyping is a quarter of the price of exome sequencing
  - ○ If so we can afford to chip genotype four times as many trios as we can afford to exome sequence, i.e. *n* = 4 × 4 = 16

then

$$ E\left[m\right]=n{v}^c=16{(0.12)}^2=0.23 $$

So for this study, given the chosen parameter values, exome sequencing is 26% better (0.29/0.23 = 1.26) than CNV calling via GWAS chip genotyping.

The Example 1 estimates and conclusion depend on the hit rates of the two assays. How human genetic variation is split between point mutations and structural changes is now quite well characterised [15]. Although fewer in number, CNVs account for more genetic variation between individuals than point mutations. Less well understood is the functional impact on disease of the various mutation classes. It has been estimated that CNVs cause transcript differences in 3% and coding differences in 1% of genes [15]. The mutation rate of CNVs [16] and point mutations [17] are such that the number of bases affected per generation via CNVs is far higher than via point mutations. Also given these mutation rates there are disproportionately fewer CNVs than point mutations implying stronger purifying selection against CNVs. Purifying selection is also suggested by a paucity of (i) common CNVs in genes, (ii) overlapping CNVs in genes, and (iii) deletion CNVs in genes and enhancers [15]. On this basis CNVs probably have larger effect sizes on traits than point mutations. We have based our estimation of the hit rate for chip genotyping for CNVs, WES and WGS assays on diagnosis rates for severe intellectual disability obtained from these assays [18]. One in 200 new born suffer severe intellectual disability (IQ < 50), and a large number of genes have been implicated. A cohort of cases were subjected to genetic testing, after each assay an attempt was made to identify a genetic cause. First they were 250 K chip genotyped for CNVs. then exome sequenced for rare point mutations, then assayed via WGS. The diagnostic rates derived for these three assays are respectively 12%, 27% and 62%. These diagnoses where predominantly based on de novo mutations and some X linked, very few where autosomal recessive [18,19]. So we can assume a disease complexity of 1 approximately applies, and so take these diagnosis rates as lower limits for the hit rates of the respective assays.

### Scenario 2: The most effective way to extend an existing study

Another scenario in which researchers commonly find themselves, is revisiting some completed study such as the above, in order to consolidate their findings, or search for new features. If this is the case, what would be the most effective way to extend the study? Two strategies present themselves

- A – apply the previously used assay to additional cases
- B – apply an additional assay to the existing dataset

Regardless of which strategy is used the result is an augmented dataset. The analysis of the initial study complicates the analysis of the augmented dataset, sequential analysis is the field of statistics addressing this problem.

The effect of these two strategies on the parameters is as follows

- Strategy A - increases *n* to *n*_*A*_, all other variables retain their original value
- Strategy B - increases *v* to *v*_*B*_, all other variables retain their original value

The effectiveness of the two strategies A and B can be evaluated via equation (3) and are respectively

6 $$ \begin{array}{l}E\left[{m}_A\right]={n}_A{v}^c\\ {}E\left[{m}_B\right]=n{v_B}^c\end{array} $$

The highest value, *E*[*m*_*A*_] or *E*[*m*_*B*_], determines the most favourable strategy.

It is also of interest to see how these strategies relate to each other. We can do this by considering the condition for which the two strategies are equivalent in terms of the expected number of completely observed disease mechanisms, i.e.

$$ E\left[{m}_A\right]=E\left[{m}_B\right] $$

Substituting expressions (6) into the above and simplifying, reduces the equality to

7 $$ \frac{n_A}{n}={\left(\frac{v_B}{v}\right)}^c $$

Again we assume there is a limited budget, this time for extending the study, and that the amount available is the same regardless of the strategy chosen. If so then

$$ \left({n}_A-n\right){P}_A=n{P}_B $$

where

- *P*_*A*_, *P*_*B*_ are the price per subject of the assays used in strategies A and B respectively

This can be combined with (3) to give the conditions under which the two strategies are equivalent, this is

8 $$ \frac{v_B}{v}={\left(\frac{P_B}{P_A}+1\right)}^{1/c} $$

Further obvious constraints apply, e.g. *v*_*B*_ ≤ 1. Thus the strategy favoured depends on the disease’s complexity and the assays’ relative prices and hit rates, and is independent of sample size.

This can be rearranged to give

9 $$ {\Delta}_{v_B}=v\left[{\left(\frac{P_B}{P_A}+1\right)}^{1/c}-1\right] $$

and

10 $$ v={\Delta}_{v_B}/\left[{\left(\frac{P_B}{P_A}+1\right)}^{1/c}-1\right] $$

where

- ${\Delta}_{v_B}={v}_B-v$ , is the minimum extra hit rate that strategy B has to confer in order to match strategy A in cost effectiveness.

In summary, the choice between strategies can be evaluated by seeing which of the equations in (2) gives the higher value. Equation (4) allows partitioning of the parameter space according to whether strategy A or B is more favourable. Equations (5) and (6), as will be seen in the following example, also allow one to assess how strongly one strategy is favoured over the other.

### Example 2

Before discussing the implications of this let us return to our CRS example. Let us compare exome sequencing of another trio (strategy A), to GWAS chip genotyping for CNV detection of the extant trios (strategy B). We will assume the same assay costs as in the previous example; these two strategies are then equivalent in cost. Complexity, and hit rate values we also take from the previous example.

Although not generally the case, we will assume no overlap between the mutations detectable via the two assays, i.e. *v*_*B*_ equals the sum of the hit rates for the two assays. Although software exists for calling CNVs from WES data, this is a difficult task due to the unevenness of WES’s enrichment stage. In our CRS study we tried calling CNVs by four different software programs [20-23] but found no consistency between their results, and concluded that CNVs cannot be reliably called using WES data. Any one of the programs could have been performing well but we cannot tell which. The only WES data available to the programs was that of our CRS dataset, it could be that given WES data on more subjects the programs might have performed better. A recent evaluation of such software drew similar conclusions [24].

Exome sequencing an additional trio increases the expected number of complete disease causing mechanisms to

$$ E\left[{m}_A\right]={n}_A{v}^c=5{(0.27)}^2=0.365 $$

Whereas chip genotyping for CNV detection our existing 4 trios, gives

$$ E\left[{m}_B\right]=n{v_B}^c=4{\left(0.27+0.12\right)}^2=0.61 $$

Clearly, given assumptions, strategy B is the better option.

**Figure 1:**
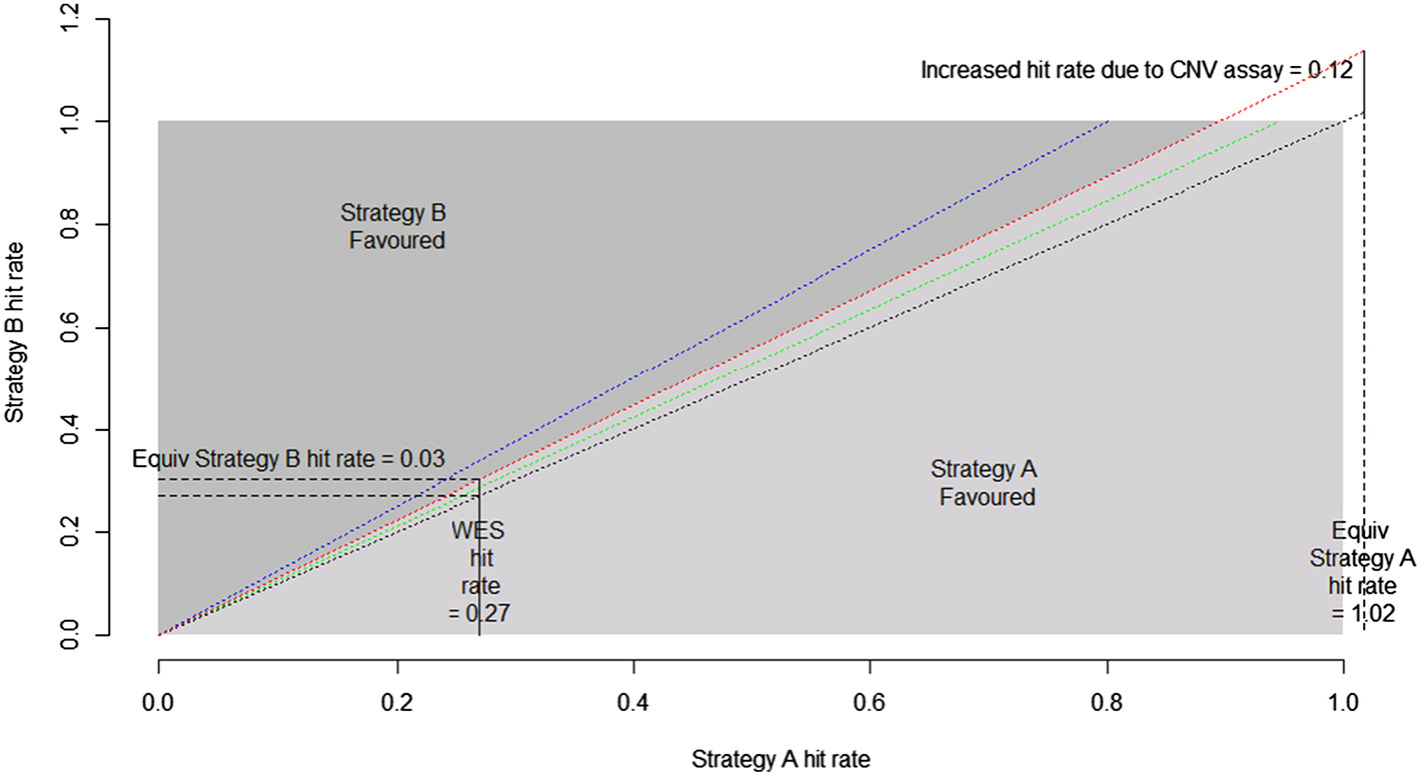
**Partitioning of the parameter space according to which strategy is favoured, in the Example 2 scenario.** Grey represents the feasible region; dark grey - strategy B favoured, light grey - strategy A favoured, given a disease complexity of 2. Blue, red, green and black dotted lines mark equivalence of the two strategies given disease complexities of 1, 2, 4 and infinity respectively. The black dotted line also represents when the new assay of strategy B provides no new information.

Also of interest is the breakeven point at which the two strategies become equally effective. If we hold the exome sequencing hit rate at 0.27 then the required ${\varDelta}_{v_B}$ as calculated by equation (5) is 0.032. Conversely equation (6) allows us to calculate the exome sequencing hit rate required for strategy A to be as good as strategy B (given ${\varDelta}_{v_B}=0.12$); that turns out to be *v* = 1.02, an impossibly high hit rate. So it seems clear that strategy B (chip genotyping of the extant trios) is better than strategy A (exome sequencing a 5^th^ CRS trio). For strategy A to even be equivalent we would need to assume an unrealistically high hit rate for exome sequencing (1.02), or unrealistically low hit rate for chip genotyping for CNV detection (0.032). The partitioning of the parameter space according to whether strategy A or B is more favoured for example 2 is illustrated in Figure 1.

## Discussion

Phenotypic variation arises from genetic and epigenetic variation in a variety of ways. The most familiar mechanism is that of a genetic mutation coding for a change in the protein’s amino acid sequence, which alters the protein’s physical or chemical properties directly affecting phenotype. Mutations acting in this way, known as non-synonymous mutations, include missense mutations, stop codon loss/gain, start codon gain, and frameshift mutations. Relatively accurate predictions can be made regarding how deleterious such mutations are because the mechanism through which they act, namely translation (of codons into amino acid sequence), is well understood. However much phenotypic variation seems driven not by changes to protein amino acid sequence but by where and when and in what quantities proteins are expressed, isoform ratios, protein folding, protein turnover and post translational modifications. In a survey of 151 GWAS papers reporting 531 SNP-trait associations, 43% were intergenic and 45% were intronic [25]. A global analysis of the control of gene expression suggested mRNA abundance explains 40% of the variation in protein levels [26]. Evidence that synonymous mutations contribute to disease, and mechanisms by which they may affect mRNA and protein levels, splicing and folding is reviewed in [27]. The control of all this is Byzantine and less well understood. Prediction of a genetic mutation’s impact on most of these processes is in its infancy. One way to overcome weakness in genetic mutation impact prediction for non-translation processes is through directly assaying other components of the machinery. Assays of the epigenome can address whether or not a region of the genome is being actively transcribed. Assays of the transcriptome can look at the quantities and sequence of mRNA and non-coding RNA involved in protein expression. The proteins themselves can be measured via mass spectrometry. These non-genetic assays are not without their own problems. Generally they are expensive, difficult to do, and often have low coverage of the class assayed. They are also, to varying degrees, dependent on the tissue type upon which they are conducted. For many diseases, biopsy of the most appropriate tissue is expensive or impractical.

Broadly speaking high throughput assays are based on two technologies; microarray and NGS. NGS is costly and analysis is computationally demanding, but all variants of a class can be assayed, including rare and *de-novo* variants. Microarray is cheaper and simpler but the variants assayed (usually a sample of the common variants) are predetermined, and *de-novo* variants are not covered. Common variants are unlikely to be major risk factors for rare disorders, but can act as modifiers of progression and phenotype. CNVs are the one class of rare and *de-novo* variation assayable using microarray technology. CNVs greater than a certain size (measured in terms of the number of consecutive assayed SNPs spanned) can be reliably called from GWAS chip intensity data. A feature of structural changes is that they can generate effects at both the source and destination loci. However CNVs identified via GWAS chip can only inform on the former. It is notable that several classes of variation are not well covered by high-throughput assays. Most classes of DNA repeats are not well covered. Another class not readily assayed are balanced structural changes, i.e. inversions and translocations.

It is clear from equation (1) that high complexity in the disease causation mechanism strongly favours using assays with a higher hit rate. If high hit rate and low sample size is desirable for complex diseases, one may then wonder why GWA studies (shallow assays of large numbers of cases and controls) have been so successful for multifactorial diseases. The explanation is to be found in a difference in underlying assumptions. In GWA studies one is looking for commonality across cases in the genetic mutation. An assumption of the study designs described here is that there is *no* overlap in the causal mutations across cases. Such commonality as there is across cases is only sought after the identification of a set of candidate disease causing mechanisms per case. Commonality across cases may then be sought in features associated with the candidate mechanisms, e.g. an implicated gene or pathway.

A question on many researchers’ minds is whether WGS is worth the considerable extra cost entailed. Our framework goes some way towards allowing such an assessment. An assay offering only a modestly increased hit rate over much cheaper rivals may still be preferable if the disease is complex enough. WGS is currently about four times dearer than WES. Given this, if the hit rate of WGS exceeded twice that of WES then it would be preferable as long as disease complexity was two or more. WGS can assay all variation assayed by WES and chip genotyping. It can see CNVs not detectable by chip genotyping. It can also detect inversions, translocations, balanced insertions/deletions and other variants invisible to the other assays. However it is hard to predict the deleteriousness of intergenic variants especially point mutations, and this reduces somewhat the apparent advantage of WGS.

The values that come out of equation (3) for *E*[*m*] are quite sobering. In our own CRS study we have at least one quite plausible candidate mechanism per case, whereas given Example 1 we should expect *E*[*m*] = 0.27. The discrepancy could be due to (i) lots of false positive disease causal mechanisms, (ii) the value of *v* is really much higher (or *c* lower) than that used in Example 1, and (iii) good luck; knowing the binomial distribution, equation (2), we can calculate how lucky we would have had to be. If the probability that all relevant risk mutations for a case have been observed is low (low value of *d*), then we should have less expectation of finding commonality across cases, and should instead concentrate on evaluating/validating our plausible candidate mechanisms, via for instance functional studies.

The framework we propose has several limitations. We assume that complete observation of a disease causing mechanism is a prerequisite for its identification, and provide a framework for maximising the number of completely observed mechanisms in the study, by maximising *E*[*m*]. However the ultimate aim is not just to have observed disease causing mechanisms but (i) to distinguish them from other plausible candidates, and/or (ii) to identify associating features (for instance an implicated gene or pathway). The ability to do either depends not only on maximising *E*[*m*] but also on minimising the number of candidate disease causing mechanisms per case.

We have not attempted to formally address this major issue, but discuss it here. In Example 1 we compared *E*[*m*] for different assays in a trio study. Using a singleton case only rather than trio study design would have trebled those *E*[*m*] estimates. However trio information allows identification of *de-novo*, compound heterozygous, and family history consistent events, and so reduces the number, and improves the quality of the candidate disease causing mechanisms found. This advantage may more than offset for the lower *E*[*m*]. Just as study design may influence quality and quantity of candidate disease causing mechanisms, so too may assay choice. For instance WGS having higher coverage than WES should have higher *E*[*m*], but predicting the deleteriousness of a mutation is more difficult outside of genic regions, so the candidate disease mechanism quality may be lower for WGS than WES. If you think that CNVs (and not point mutations) are the only intergenic mutations for which you can have some confidence of major effects then you may be tempted to use chip genotyping for CNV detection as a cheaper alternative to WGS. Having said that, more CNVs can be detected by WGS than chip genotyping (see [18]), and translocations and inversions can also be detected.

Error rate differs between assays and this may also affect the number of candidate disease mechanisms generated. It also changes the costs that need to be considered. The cost of validating the candidate disease mechanisms an assay produces should be taken into account when choosing between assays.

Disease aetiology has a major impact on the effectiveness of a study design, however typically the aetiology will be largely unknown to us. Study power is sensitive to the complexity parameter *c*, a characteristic of the disease. The number of plausible disease causing mechanisms that can be constructed per case, explodes exponentially with increasing *c*, with a commensurate reduction in study power. Thus we may set complexity to a low value, e.g. *c* = 2, on the basis that our study would have no power for high levels of disease complexity. A similar rationale lies behind the common practice of disregarding common variants in rare disease aetiology research, namely doing so reduces the number of candidate disease mechanisms generated.

Another limitation of our framework is its dependence on parameter values. Disease complexity has already been discussed. The difficulty in setting the hit rate for any particular assay should be apparent from the examples. Although there is information relating to how human genetic variation is split between the various classes (e.g. point mutations, structural changes), less well understood is how disease risk mutations are split between the various classes of mutation. Despite this difficulty it is apparent from Example 2 that even given poor parameter estimates it still seems possible to make informed decisions about assay choice.

Despite its shortcomings our framework provides some practical guidance as to the selection of assays when looking for genetic risk factors for rare diseases. It helps make explicit the relationships between many of the variables involved. It also exposes where current knowledge is lacking and points the way to future research directions. The implications the framework generates bring to mind the Indian tale of the blind men describing an elephant. It seems assaying of each class of mutation/variation adds a dimension, aiding comprehension, and that deeply assaying a few individuals should be more fruitful than the shallow assaying of many.

## Abbreviations

NGS: Next generation sequencing
WGS: Whole genome sequencing
WES: Whole exome sequencing
WS: Waardenburg syndrome
CRS: Caudal Regression Syndrome
VACTERL: Association of vertebral anomalies, anal atresia, cardiac defects, Tracheoesophageal fistula and/or esophageal atresia, renal & radial anomalies and limb defects
GWA: Genome Wide Association
GWAS: Genome Wide Association Study
CNV: Copy number variant
SNP: Single nucleotide polymorphism
mRNA: Messenger ribonucleic acid
DNA: Deoxyribonucleic acid
